# Draft Genome Sequence of Clostridium botulinum B2 450 Strain from Wound Botulism in a Drug User in Italy

**DOI:** 10.1128/genomeA.00238-15

**Published:** 2015-04-02

**Authors:** Silvia Fillo, Francesco Giordani, Anna Anselmo, Antonella Fortunato, Anna Maria Palozzi, Riccardo De Santis, Andrea Ciammaruconi, Ferdinando Spagnolo, Fabrizio Anniballi, Alfonsina Fiore, Bruna Auricchio, Dario De Medici, Florigio Lista

**Affiliations:** a Histology and Molecular Biology Section, Army Medical and Veterinary Research Center, Rome, Italy; b National Reference Center for Botulism, Department of Veterinary Public Health and Food Safety, Istituto Superiore di Sanità (ISS), Rome, Italy

## Abstract

Here, we report the draft genome sequence of Clostridium botulinum B2 450, responsible for the first reported case of wound botulism in a drug user in Italy.

## GENOME ANNOUNCEMENT

Clostridium botulinum is a microorganism able to produce the botulinum neurotoxin (BoNT), a powerful poison that causes botulism, a serious neuroparalytic disease. There are 3 mainly clinical manifestations of botulism: foodborne (ingestion of BoNT-contaminated foods), intestinal (BoNT is produced by intestinal colonization of C. botulinum), and wound (C. botulinum spores germinate and synthesize BoNT in contaminated wounds) (1). Based on its physiological characteristics, C. botulinum is divided into four different groups (I to IV), so phylogenetically different that they can be considered separate species (2, 3). Moreover, other species, such as Clostridium baratii and Clostridium butyricum, are able to produce botulinum neurotoxins type F and E, respectively. The BoNT is classified by 8 serotypes (A to H), each of which is divided into subtypes. To date, 16 full genomes and several draft assemblies of C. botulinum are available, prevalently isolated from food borne and infant cases. A5(B3′)H04402 065 (accession no. FR773526) is the only full genome sequence originating from a wound botulism case (4, 5).

The B2 450 strain was isolated in 2009 from wound exudate of a heroin user patient, in Messina, Sicily, Italy (6). Sequencing was performed using both Roche 454 GS FLX Titanium and Illumina MiSeq platforms. Roche sequencing generated 286,392 single-end reads, with 112,174,234 total sequenced bases, ~28-fold coverage. The reads were *de novo* assembled with the GS Assembler software (Newbler package) into 160 contigs, and 2,430,881 paired-end reads were produced by Illumina sequencing with 731,610,908 total sequenced bases and ~185-fold coverage. Illumina reads were assembled with Abyss-pe producing 497 contigs. 454 and Miseq contigs were combined using Minimus2 software, and 18 contigs were obtained.

A well-known flaw of the 454 platform is the erroneous determination of homopolymer lengths (7); 1,218 homopolymeric stretches were corrected according to Illumina sequences. Nine gaps were closed using 454 and Miseq reads or Sanger sequencing.

The final draft assembly consists of 9 chromosomal contigs, for a total length of 4,070,655 bp, and one plasmidic contig, 250,014 bp long, containing the BoNT/B2 gene. The genome has a G+C content of 27.8%. The 8 remaining gaps are due to repeated sequences (rRNA operon and beta-*N*-acetyl-glucosamidase genes).

The 16S rRNA gene sequence of B2 450 belongs to group I showing 99.8% similarity with the A1 ATCC 3502 16S sequence (NC_009495) (3). Comparing some gene sequences (rpoB-mdh-aroE-hsp60-aceK-oppB-recA) (8), the B2 450 strain appears phylogenetically closer to Clostridium sporogenes, with 99.99% similarity to ATCC 15579 (ABKW0200000), than to all other C. botulinum strains (96.14% to A1 ATCC 3502 and 95.79% to A3 Loch Maree; NC_010520). C. sporogenes phylogenetically belongs to group I (9). The 450 strain may represent a C. sporogenes lineage that has recently acquired the BoNT/B gene (perhaps through the plasmid) (10).

### Nucleotide sequence accession number.

The genome sequence of C. botulinum B2 450 was deposited at DDBJ/EMBL/GenBank under the accession no. JXSU00000000. The version described in this paper is the first version.
